# TrypOx, a Novel Eukaryotic Homolog of the Redox-Regulated Chaperone Hsp33 in *Trypanosoma brucei*

**DOI:** 10.3389/fmicb.2020.01844

**Published:** 2020-08-06

**Authors:** Samar Aramin, Rosi Fassler, Vaibhav Chikne, Mor Goldenberg, Tal Arian, Liat Kolet Eliaz, Oded Rimon, Oren Ram, Shulamit Michaeli, Dana Reichmann

**Affiliations:** ^1^Department of Biological Chemistry, The Alexander Silberman Institute of Life Sciences, Safra Campus Givat Ram, The Hebrew University of Jerusalem, Jerusalem, Israel; ^2^The Mina and Everard Goodman Faculty of Life Sciences, Advanced Materials and Nanotechnology Institute, Bar-Ilan University, Ramat Gan, Israel

**Keywords:** Hsp33, redox regulation, redox regulation in *T. brucei*, stress-regulated chaperones, holdase chaperone

## Abstract

ATP-independent chaperones are widespread across all domains of life and serve as the first line of defense during protein unfolding stresses. One of the known crucial chaperones for bacterial survival in a hostile environment (e.g., heat and oxidative stress) is the highly conserved, redox-regulated ATP-independent bacterial chaperone Hsp33. Using a bioinformatic analysis, we describe novel eukaryotic homologs of Hsp33 identified in eukaryotic pathogens belonging to the kinetoplastids, a family responsible for lethal human diseases such as Chagas disease as caused by *Trypanosoma cruzi*, African sleeping sickness caused by *Trypanosoma brucei* spp., and leishmaniasis pathologies delivered by various *Leishmania* species. During their pathogenic life cycle, kinetoplastids need to cope with elevated temperatures and oxidative stress, the same conditions which convert Hsp33 into a powerful chaperone in bacteria, thus preventing aggregation of a wide range of misfolded proteins. Here, we focused on a functional characterization of the Hsp33 homolog in one of the members of the kinetoplastid family, *T. brucei*, (Tb927.6.2630), which we have named TrypOx. RNAi silencing of TrypOx led to a significant decrease in the survival of *T. brucei* under mild oxidative stress conditions, implying a protective role of TrypOx during the Trypanosomes growth. We then adopted a proteomics-driven approach to investigate the role of TrypOx in defining the oxidative stress response. Depletion of TrypOx significantly altered the abundance of proteins mediating redox homeostasis, linking TrypOx with the antioxidant system. Using biochemical approaches, we identified the redox-switch domain of TrypOx, showing its modularity and oxidation-dependent structural plasticity. Kinetoplastid parasites such as *T. brucei* need to cope with high levels of oxidants produced by the innate immune system, such that parasite-specific antioxidant proteins like TrypOx – which are depleted in mammals – are highly promising candidates for drug targeting.

## Introduction

The Kinetoplastids are responsible for lethal human diseases such as Chagas disease caused by *Trypanosoma cruzi*, African sleeping sickness, caused by *Trypanosoma brucei* spp. and leishmaniasis pathologies delivered by various *Leishmania species* (Fevre et al., 2008; Schmunis and Yadon, 2010; Simarro et al., 2012). According to WHO, these diseases affect over 20 million people a year, along with continuous infection of wild and domesticated animals, which may have a serious economic impact on many countries around the world.

These parasitic pathogens are transmitted by insect vectors into mammalian blood, where they encounter oxidative stress applied by the immune system (Beschin et al., 2014; Cnops et al., 2015). Therefore, parasite survival and its successful proliferation highly depend on the capacity of the pathogen to evade immune attack and maintain its redox balance during its life cycle. Kinetoplastids have developed multiple strategies including life-cycle-dependent changes in respiration and mitochondrial morphology, as well as synthesis of highly efficient antioxidants and molecular chaperones, protecting the pathogen’s proteome during unfolding conditions (Krauth-Siegel et al., 2003; Folgueira and Requena, 2007; Tyedmers et al., 2010). Members of this network have been potential drug targets over the last 10 years (Leroux and Krauth-Siegel, 2016). However, efforts to generate an efficient treatment to combat the serious diseases caused by these parasites have failed.

The antioxidant machinery of trypanosomes heavily relies on the reduced form of the dithiol trypanothione, serving as one of its most efficient antioxidants (Muller et al., 2003; Castro and Tomas, 2008; Piacenza et al., 2013). Consequently, the pathogen’s antioxidant enzymes are evolved to use trypanothione as a cofactor for detoxification of endogenous reactive oxygen and nitrogen species, as well as restoring the redox status of proteins thiols (Krauth-Siegel et al., 2007; Castro and Tomas, 2008; Beltrame-Botelho et al., 2016).

Accumulation of oxidants causes substantial protein damage, which can lead to widespread oxidative protein unfolding, aggregation, and ultimately cell death. Therefore, in addition to evolving a repertoire of antioxidants, kinetoplastids have a highly regulated and dynamic chaperone network, including very-well conserved ATP-dependent chaperones Hsp70, Hsp60, and Hsp40, ATP-independent small heat shock proteins (sHSP) (Hombach et al., 2014; Perez-Morales and Espinoza, 2015; Costa-Martins et al., 2018; Anas et al., 2019), and members of protein degradation machinery (Anas et al., 2019) families that may play a key role in trypanosome biology (Haslbeck et al., 2005; Folgueira and Requena, 2007; Louw et al., 2010). However, the detailed mechanism of these chaperone networks is not yet clear, nor are their potential substrates, regulation, and role in protozoa development or adaptation to new environmental conditions understood.

One of the known crucial chaperones for pathogens’ survival in a hostile environment is the highly conserved, redox-regulated ATP-independent bacterial chaperone Hsp33 (Jakob et al., 1999; Kang et al., 2007; Winter et al., 2008; Wholey and Jakob, 2012; Suss and Reichmann, 2015; Krewing et al., 2019). Hsp33 is rapidly activated by site-specific oxidation, and serves as a first line of defense during particularly problematic stress conditions that lead to widespread protein unfolding (Winter et al., 2008; Cremers et al., 2010; Rimon et al., 2017). Oxidation-induced activation of Hsp33 compensates for ATP depletion that inactivates canonical chaperones during oxidative stress, and makes Hsp33 one of the central members of the cellular antioxidant defense system (Winter et al., 2005; Reichmann et al., 2018). Under non-stress, reduced, conditions, Hsp33 is a compactly folded zinc binding protein with negligible chaperone activity. However, oxidation of Hsp33 promotes massive conformational rearrangements which activate its anti-aggregation function (Supplementary Figure S1A) (Ilbert et al., 2007; Cremers et al., 2010; Reichmann et al., 2012; Lee et al., 2015; Suss and Reichmann, 2015; Groitl et al., 2016). These changes occur via oxidation of four highly conserved cysteine residues located in Hsp33’s C-terminal redox switch domain (Graf et al., 2004; Enescu et al., 2015). Triggered by disulfide bond formation and zinc release, the redox switch domain of Hsp33 unfolds, leading to further unraveling of the adjacent linker domain and exposure of its hydrophobic surfaces, which are involved in recognition of the aggregation-prone client proteins (Ilbert et al., 2007; Groitl et al., 2016). Upon return to normal, reducing conditions, the cysteine thiols are restored, which leads to refolding of Hsp33, destabilization of the bound client protein and its transfer to a canonical bacterial chaperone system, DnaK/J (e.g., Hsp70/Hsp40 in eukaryotes), for further refolding (Ilbert et al., 2007; Reichmann et al., 2012). It was also suggested that Hsp33 has a critical role in promoting the folding process of the misfolded client protein using its structural plasticity (Moayed et al., 2020).

Here, we utilized bioinformatic analysis and revealed several eukaryotic Hsp33 homologs spanning many different taxonomical families, including the kinetoplastids (i.e., *Trypanosoma* and *Leishmania*), unicellular algae, and higher plants, with a majority of them found in kinetoplastids. In this study, we focused on characterization of the Hsp33 homolog in *Trypanosoma brucei* (Tb927.6.2630), which we have named TrypOx. We showed that RNAi silencing of TrypOx led to significant decreases in the survival of *T. brucei* under mild stress conditions, implying a protective role for TrypOx. We adopted a proteomics-driven approach to investigate the role of TrypOx in defining the oxidative stress response and found a significant decrease in the abundance of proteins mediating redox homeostasis upon TrypOx depletion. Using biochemical approaches, we identified the redox-switch domain of TrypOx, showing its modularity and oxidation-dependent structural plasticity.

## Results

### The Hsp33 Family Is Highly Conserved Among Prokaryotes and Kinetoplastids

**FIGURE 1:**
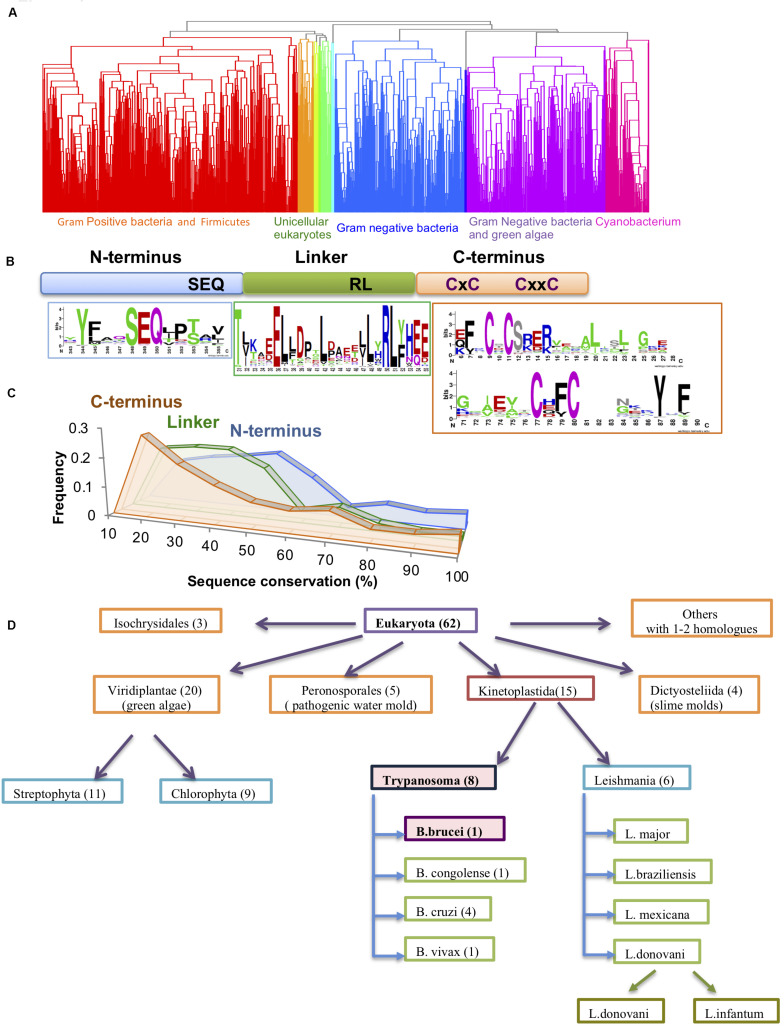
Conservation of the Hsp33 protein family. **(A)** Clustering of the 1579 Hsp33 homologs according to their sequence conservation derived by hierarchical clustering as described in the method part. Ten obtained clusters (shown in different colors) correspond to the phylogenetic origin of the sequences. **(B)** Schematic representation of the Hsp33 domains. Sequence motifs of the most conserved sequence regions are shown in sequence logos created by WebLogo (Crooks et al., 2004). **(C)** Comparison of sequence conservation between the three Hsp33 regions [N-terminus (blue), linker (green) and C-terminal redox domain (orange)]. The plot shows the distribution of the position-based amino acid conservation scores as described in section “Materials and Methods.” **(D)** Hsp33 orthologs in eukaryotes. Kinetoplastida and Viridiplantae families contain the larger numbers of Hsp33 orthologs.

Hsp33 is a first line of defense chaperone, protecting organisms ranging from bacteria (Jakob et al., 1999; Wholey and Jakob, 2012) to green algae (Segal and Shapira, 2015) against the toxic effects of oxidative stress. By conducting a PSI-BLAST analysis, we identified 1579 homologous sequences with identity scores below 85. While the majority of sequences were found in prokaryotes, we identified ∼70 representatives in eukaryotic species, mainly in unicellular parasites (e.g., *Trypanosoma, Leishmania, Phytophthora, Naegleria*, etc.), unicellular organisms characteristically found in extreme environmental habitats (e.g., *Galdieria*, *Phaeodactylum*), and a few higher plants (e.g., *Oriza, Ricinus*) (Figure 1A). Thus, it is tempting to speculate that Hsp33 might be an important factor that allows these organisms to endure their hostile living environments, particularly for pathogens, as these must cope with the adamant and offensive response set off by the host immune system.

Intriguingly, with the exception of a few plant members, *Archaea* and multicellular eukaryotes lack the Hsp33 homolog and it is likely that other chaperones in these species fulfill its role instead.

The Hsp33 sequence comprises a well-structured N-terminal domain (*E. coli*: 1-174aa) and a highly flexible C-terminal domain (*E. coli*: 227-292aa) (Supplementary Figure S1A). The latter acts as a redox sensor via oxidation of its four fully conserved cysteine residues (Figure 1B, orange)(Won et al., 2004; Leichert et al., 2008), which leads to destabilization of the linker region and unfolding of the C-terminal domain (Supplementary Figure S1A). The two domains are connected by a metastable linker region (Figure 1B, green). It has been shown experimentally that the C-terminal domain can serve as an independent redox-regulated module and may be fused to a non-related functional domain (e.g., Protein Kinase C domain) (Zhao et al., 2011). To further examine the modularity of the Hsp33 domains, we divided the homologous sequences into three fragments based on a multiple sequence alignment in which the *E. coli* Hsp33 sequence served as reference (see “Materials and Methods” section). Sequences from these three blocks were then used as the input for additional runs of PSI-BLAST analysis. To our surprise, we did not detect any proteins harboring only one or two of the three Hsp33 domains, suggesting that there is a restricted functional modularity of the complete set. We did identify a small subset of proteins with additional functional domains fused to the Hsp33 protein (Supplementary Figure S1B). These include eleven unique bacterial Hsp33 proteins from the *Mycoplasma* species, which have a C-terminally fused CinA domain, thought to be involved in bacterial competence and transformation (Martin et al., 1995). Moreover, 31 multiple species of the gram negative *Thermus* bacteria harbor a unique Zn-ribbon motif adjusted to the Hsp33 Zn motif, at its C-terminus. At this stage, it is not clear if multiplication of the Zn binding domains leads to increased affinity of Zn (or other metal) binding, providing a new mode of regulation. Interestingly, eukaryotic unicellular plant pathogens harbor either an N-terminal BTB/POZ domain crucial for transcription, ubiquitination, and protein interactions (Furukawa and Xiong, 2005) or a kinesin associated protein domain (KAS) usually found in the motor protein kinesisn-2 (Supplementary Table S1, Supplementary Figure S1B).

Sequence comparisons indicated a low sequence similarity in the linker region (average conservation of 35%) and the C-terminal domains (average conservation of 30%) (Figures 1B,C), with more than one third of all sequences falling below the similarity threshold (30% identity and *e*-value < 0.001) (Figure 1C). This result suggests that the conservation of the Hsp33 protein family resides mainly within the N-terminal domain, as well as the highly conserved cysteine motif comprised of 4 cysteines (CxC and CxxC motifs) in the C-terminal redox switch domain (Figure 1B). In fewer than 10 out of 1579 sequences, the second cysteine was not conserved. In contrast, only two very short motifs were detected in the 50-aa linker region of the Hsp33 homologs, comprising of the conserved residues TxxxxE and RL(Y/F) (Figure 1B).

The sequence analysis revealed high conservation of Hsp33 in the Kinetaplastida family, which includes both *Trypanosoma* and *Leishmania* species (Figure 1D), pointing to a potential functional role for Hsp33 in these species. Puzzled by Hsp33’s potential importance in trypanosome species, we decided to focus on the *T. brucei* Hsp33 homolog (Tb927.6.2630), which we have named TrypOx.

### TrypOx Is Crucial for the Survival of *T. brucei* During Mild Oxidative Stress Conditions

It is known from previous research that Hsp33 is crucial for bacterial survival during oxidative stress (Jakob et al., 1999), reflecting one of the bacterial strategies to cope with the host’s immune system attacks, which uses oxidants (e.g., H_2_O_2_) to kill pathogens. In light of Hsp33’s important role in the bacterial defense, it was tempting to assume that TrypOx, being an Hsp33 homolog, would have the same defensive role.

**FIGURE 2:**
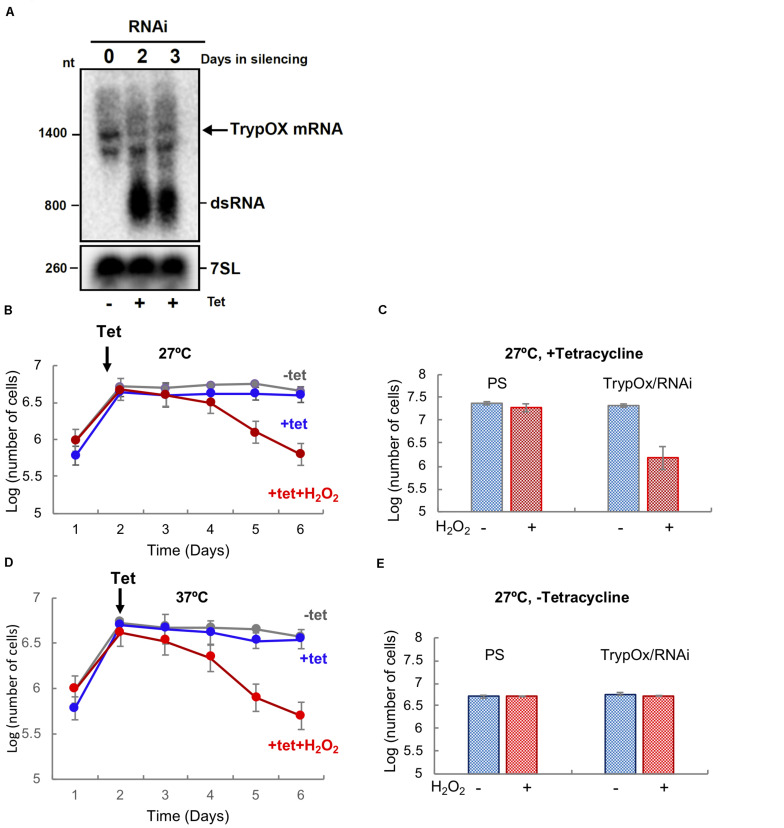
TrypOx is crucial for the survival of *T. brucei* during mild oxidative stress conditions. **(A)** Northern blot analysis of RNA from the parental *T. brucei* 29-13 cell line was compared with RNA from the *T. brucei* clones with RNAi TrypOx induced with tetracycline (+Tet) for 3 days. The blots were treated with the *T. brucei* 7SL gene as the control. Successful knockdown was observed in clones 2, 3, and 5. **(B,C)** Survival of *T. brucei* cells in the presence of tetracycline-induced TrypOx RNAi in normal (blue) or mild oxidative stress (red) (50 μM H_2_O_2_) at 27°C **(B)** and 37°C **(C)**. Non-induced cells were used as a control (in gray). Tetracycline was added at day 2 after dilution of the cells to 10^6^ cells. **(D,E)** Changes in the number of induced and non-induced cells in the presence or absence of H_2_O_2_. Corresponding to day 5 in panel **B,C**). The standard deviation of triplicate and quadruplicates counts are indicated by error bars.

In order to test our hypothesis, TrypOx expression was knocked down by RNAi using the tetracycline-induced synthesis of small interfering RNA fragments (siRNA) derived from expression of long dsRNA. Silencing of the TrypOx mRNA synthesis was validated by Northern analysis in different clones, before and after RNAi induction at 1–3 days after the induction (Figure 2A). The *T. brucei* 7SL RNA was used as a loading control. Growth of induced and non-induced cells was monitored after daily dilution to 10^6^ cells/ml. Growth was monitored for 5 days in the absence and presence of 50 μM H_2_O_2_ (Figures 2B–E, Supplementary Figure S2). The depletion of TrypOx resulted in significant growth inhibition upon mild oxidative stress induced by H_2_O_2_ (Figures 2B–D, Supplementary Figures S2C,D), while survival of the wild type parental strain was not affected (Figures 2D,E, Supplementary Figure S2B). This growth decrease was mainly observed after 3 days of silencing and was found to be temperature independent (Figures 2B,C, Supplementary Figures S2C,D). Similar results were observed with additional TrypOx RNAi clones, pointing to the significance of TrypOx in survival during exposure to non-toxic concentrations of peroxide. These results are in line with those obtained in the *E. coli* and *V. cholera* Hsp33 (*hslo*) knockout strains, which also leads to bacterial sensitivity to oxidative stress conditions (Jakob et al., 1999; Winter et al., 2005; Wholey and Jakob, 2012). Interestingly, in bacteria, depletion of Hsp33 led to growth deficiency in more severe conditions (oxidation coupled with unfolding conditions, e.g., HOCl or peroxide and heat shock) (Jakob et al., 1999; Winter et al., 2008; Wholey and Jakob, 2012) then in *T. brucei*.

### TrypOx Knockdown Alters Abundance of Redox-Related Proteins in *T. brucei*

**FIGURE 3:**
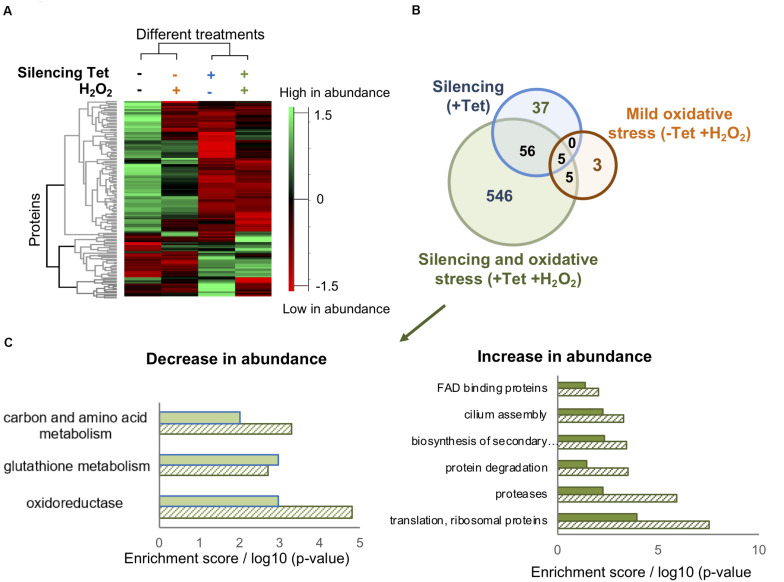
TrypOx silencing alters the *T. brucei* proteome. **(A)** Hierarchical clustering of the median expression values of all differentially expressed proteins (FDR < 0.05) identified in the four differently treated TrypOx RNAi cells **(B)** Venn diagram of the proteins with significantly different abundance (FDR < 0.05) relative to protein abundances in the non-treated cells **(C)** Functional enrichment analysis (filled bars) of the 546 differentially expressed proteins in induced RNAi TrypOx cells treated with 50 μM peroxide and the Benjamini *p*-Value for each group, representing the significance (slashed bars).

Intrigued by the possible crucial role of TrypOx in *T. brucei* survival during oxidative stress, we conducted comparative proteomics to identify potential key proteins affected by knockdown of TrypOx. Non-induced and induced *T. brucei* TrypOx RNAi cell lines (3-day inductions) were supplemented with either PBS or non-toxic peroxide concentration (50 μM), for 3 h, using four biological replicates for proteomic analysis. 10^8^ *T. brucei* cells from 16 samples were harvested and lysed, followed by protein extraction, trypsin digestions, alkylation, and analysis by LC-MS/MS. To gain insights into the global changes in the proteomic profile of all 16 samples, we applied label free quantification (LFQ) using the MaxQuant software (Cox and Mann, 2008) to compare the abundance of proteins in four different sample groups (treated and untreated, RNAi induced and non-induced cells). At first, we confirmed that the silencing of TrypOx resulted in no production of the TrypOx protein (Supplementary Figure S3). Moreover, we identified 4161 proteins across all 16 samples, 2046 of which were expressed in all four sample groups (Supplementary Table S2). Hierarchical clustering of differentially expressed proteins as defined by an ANOVA test showed that depletion of TrypOx leads to changes in the proteome, a difference that grew significantly upon treatment with peroxide (Figure 3A). To identify potential key proteins mediating the sensitivity of TrypOx RNAi cells during mild oxidative stress, we applied a stringent two tailed student T-test analysis (FDR < 0.05) to compare protein abundance in RNAi induced, peroxide treated induced, and non-induced cells relative to untreated, non-induced cells. This analysis identified 602 proteins showing a statistically significant different abundance relative to the untreated cells, 546 of which changed only after both silencing and peroxide treatment [Figure 3B (green circle in the Venn diagram), Supplementary Table S3]. This suggests that these proteins are associated with the oxidation-dependent growth defect following TrypOx depletion. A functional enrichment analysis of 104 proteins with decreased abundance clearly pointed to the role of the redox homeostasis system in the growth decline. Specifically, peroxide treatment of TrypOx-depleted cells led to a significant change in levels of different redox-regulated proteins. These include Glutaredoxin (Grx), Glutaredoxin peroxidase, ribonucleoside-diphosphate reductase (which restores oxidized thiols of thioredoxin and mediates glutathione metabolism), and oxidoreductases producing one of the major reducing cofactors NADH (pteridine reductase, mitochondrial malate dehydrogenase, isocitrate dehydrogenase, prostaglandin synthase and others (Supplementary Table S3, Figure 3C). Analysis of the subset of proteins with increased abundance demonstrates an increase in the Tryparedoxin peroxidase, (TXNPx, Tb927.8.1990), one of the key defense proteins in *T. brucei* during oxidative stress. TXNPx protein level was significantly decreased upon peroxide treatment in general, regardless of the TrypOx depletion, in line with previously published studies (Budde et al., 2003). However, only cells with reduced TrypOx levels were sensitive to oxidative stress. Therefore, we may suggest that a lack of TrypOx in cells alters the efficiency of peroxide detoxification, which might be one of the reasons for the growth defect. However, despite multiple attempts to measure precise changes in peroxide and GSH/GSSH levels in TrypOx RNAi cells, the results were inconsistent, varying between different commercial kits and conditions. Therefore, at this point, we are not able to say whether TrypOx modifies endogenous levels of oxidants.

Other redox-regulated proteins such as trypanothione reductase (Tb10.406.0520) and trypanothione synthase (Tb927.2.4370) were identified in our study, however, their abundance was unaffected by any of the treatments (TrypOx inactivation or peroxide treatment), suggesting that cells are not under heavy stress.

One of the hallmarks of oxidative stress is an accumulation of ribosomes and an increase in protein degradation (Reichmann et al., 2018; Shcherbik and Pestov, 2019). Induction of TrypOx RNAi in the presence of peroxide led to a significant increase in both ribosomal proteins and the protein degradation machinery, including various types of proteases (different classes of aminopeptidases, carboxypeptidase, cysteine peptidase, surface protease g63, and ATP-dependent Clp protease, disaggregase Hsp78 (Supplementary Table S3). This might link redox and protein homeostasis to TrypOx function in cells.

### Designing Chimeric Proteins to Investigate the Redox Regulation of the TrypOx Protein

To examine the redox regulation function of TrypOx, we decided to utilize Hsp33’s modular feature and produce a chimeric protein, comprising domains of *E. coli* Hsp33 and *T. brucei* TrypOx proteins. This followed numerous unsuccessful attempts to obtain a soluble and thermodynamically stable, full-length TrypOx protein. These attempts included the use of bacterial and eukaryotic expression systems, purification from inclusion bodies, and codon optimization, yet yielded no soluble full-length protein despite extensive effort (Supplementary Figure S4).

**FIGURE 4:**
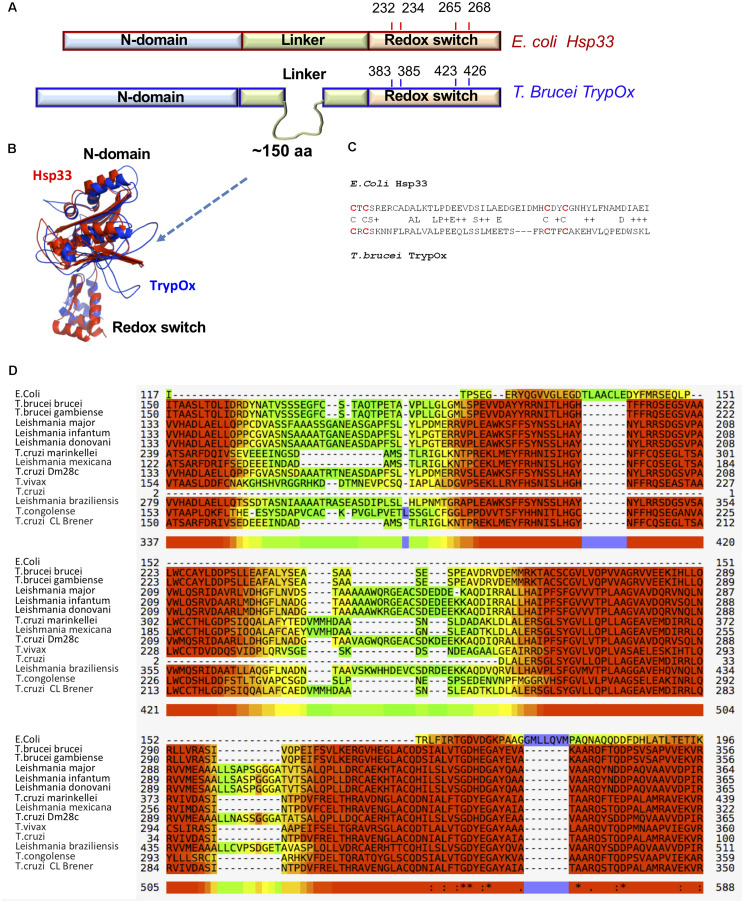
Sequence and structural similarity of TrypOx and *E. coli* Hsp33. **(A)** Schematic representation of a sequence alignment between *E. coli* (red) and *T. brucei* (blue) orthologs. The alignment points to the extension of the linker domain in the *T. brucei* ortholog. The position of the conserved cysteines (oxidation of which triggers chaperone activity in bacterial Hsp33) is shown. **(B)** Structural alignment of the TrypOx model (blue) with reduced B. subtilis Hsp33 (PDB ID:1VZY) shows high conservation of the N-terminal and C-terminal domains. **(C)** Sequence alignment of the C-terminal redox domain from *E. coli* and *T. brucei* orthologs. The redox-sensitive cysteines are in red. **(D)** Multiple alignment of the unique linker sequence Hsp33 homologs from *Trypanosoma* and *Leishmania* species aligned with *E. coli* Hsp33. The alignment was done by MUSCLE (https://www.ebi.ac.uk/Tools/msa/muscle/), color code: red to yellow, indicating high to low sequence of conservation.

To investigate domain conservation and define domain boundaries, we utilized an available and highly similar to *E. coli* Hsp33 X-ray structure of *B. Subtilis* Hsp33 (PDB:1VZY) (Janda et al., 2004) to derive a structural model of TrypOx using the MODELER software (Webb and Sali, 2016). The structural alignment between bacterial Hsp33 and a predicted model of TrypOx suggests a high structural similarity, specifically in the N-terminal and C-terminal domains (Figure 4). Interestingly, the linker region is very different between the bacterial and eukaryotic Hsp33 homologs, wherein the *Trypanosoma* and *Leishmania* Hsp33 homolog have an additional segment of ∼150aa not found in bacteria which may be functionally important in protozoa (Figure 4D). We speculate that this region was the reason for the poor solubility of the *T. brucei* homolog. To overcome the solubility problem and to investigate the modularity of TrypOx, we designed several chimeric proteins, fusing the N-terminal and linker domains with the C-terminal domains from different species (Supplementary Figure S4). All chimeric variants harboring the fused *T. brucei* N-terminal and linker domains resulted in insoluble proteins. However, replacement of the bacterial C-terminus with the TrypOx C-terminus produced a highly expressed and soluble protein.

Interestingly, the C-terminal domains of the two homologs show low sequence identity of 25%. However, both contain four conserved cysteine residues, oxidation of which leads to the partial unfolding of Hsp33 and exposure of hydrophobic regions crucial for the anti-aggregation activity (Ilbert et al., 2007; Rimon et al., 2017).

This domain has previously been shown to have functional modularity, following fusion to a non-related Protein kinase C (PKC) domain after which its redox status defined the kinase activity of PKC (Zhao et al., 2011). Therefore, we hypothesized that fusion of TrypOx’s C-terminal domain with the *E. coli* N-terminal and linker region fragments would allow us to investigate the redox-regulation of this domain and its role in maintaining the chaperone activity of Hsp33.

### The C-Terminus of TrypOx Is a Modular, Redox-Switch Domain

Hsp33 occupies two main functions: it prevents the aggregation of misfolded proteins during oxidative stress and transfers unfolded substrates to the relevant foldase system (DnaK/J-GrpE) upon return to non-stress conditions. Whereas the first function depends on the oxidation of Hsp33’s C-terminal cysteines, which triggers the exposure of the client binding sites, client transfer to the foldase system requires reduction of Hsp33’s cysteines, leading to refolding of the redox switch and the linker domains (Hoffmann et al., 2004; Ilbert et al., 2007).

To test the robustness and functional conservation of the *T. brucei* redox domain, we expressed the *E. coli* Hsp33 wild type and the designed chimeric protein (Hsp33-TrypOx) in a BL21 *E. coli* strain lacking the endogenous Hsp33 gene (BL21Δ*hslo*). Expression of the chimeric and wild type proteins complemented the oxidative stress sensitivity, induced by 20 μM HOCl, of the Hsp33 deletion (Δ*hslo)* mutant (Supplementary Figure S5), suggesting similarity in redox-related function.

The wild type *E. coli* Hsp33 and the chimeric protein, Hsp33-TrypOx, were purified as described in the “Materials and Methods” section and tested for their redox-regulated activity. The wild type and chimeric proteins were first reduced (Hsp33_red_, Hsp33-TrypOx_red_) by the reducing agent DTT and then oxidized using H_2_O_2_ under unfolding conditions (43°C) (Hsp33_ox_, Hsp33-TrypOx_ox_) as described in Fassler et al. (2018). The oxidation of the protein thiols was verified by using Ellman’s reagent (DNTB, 5,5′-dithio-2-nitrobenzoic acid).

**FIGURE 5:**
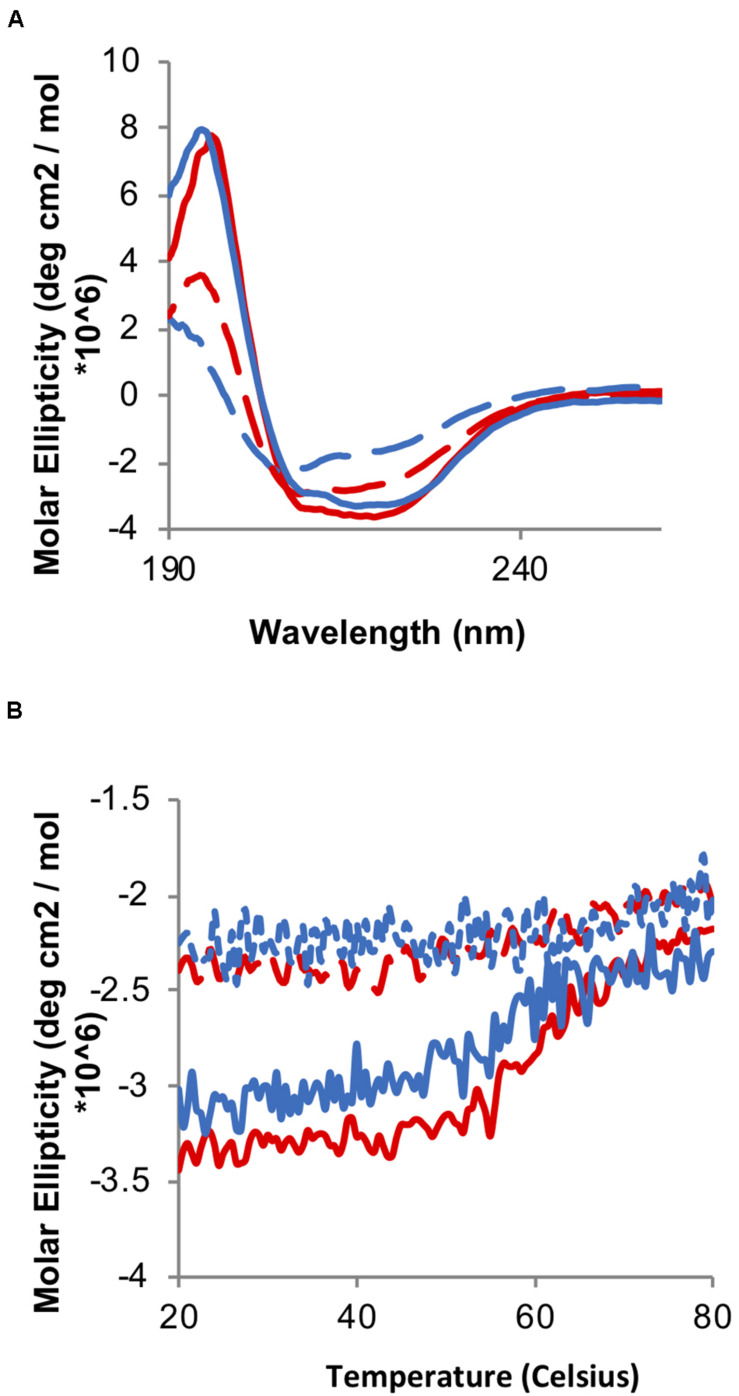
Oxidation-induced unfolding of the wild type and Hsp33-TrypOx variant. **(A)** Far-UV CD spectra of inactive reduced (solid line) and active oxidized (dashed line) wild type Hsp33 (red) and Hsp33-TrypOx proteins (blue) (5 μM). **(B)** Thermal stability of wild type Hsp33 (red) and Hsp33-TrypOx (blue) in either reduced (solid) or oxidized (dashed) forms was determined by monitoring the changes in molecular ellipticity at 217 nm. The proteins were heated from 20 to 80°C at 1°C/min.

Chaperone activity is triggered by the formation of disulfide bonds in the redox domain, leading to overall chaperone unfolding and exposing hydrophobic regions involved in binding the client proteins. Therefore, we initially decided to test whether the oxidation of the TrypOx redox domain is able to mediate Hsp33 unfolding and its chaperone activity. We applied far-UV circular dichroism (CD) spectroscopy to analyze oxidation dependent changes in the secondary structures of the Hsp33 wild type and Hsp33-TrypOx variants in their reduced and oxidized forms (Figure 5). As expected, the *E. coli* Hsp33 and Hsp33-TrypOx proteins unfold in the active, oxidized form, leading to a decrease in chirality and a shift in the circular dichroism spectrum, mainly at the helical regions (195 and 210–220 nm) (Figure 5A, red traces) comprising of the C-terminal and linker regions in the inactive, reduced form of Hsp33 (Supplementary Figure S1). To examine the thermostability of the Hsp33 wild type and Hsp33-TrypOx variants, we monitored the CD signal at 217 nm as a function of temperature. As previously described, the Tm of the wild type Hsp33 protein shifts in correspondence with its redox status (Tm of ∼60°C in the reduced form, and 40°C in the oxidized form). As expected, the Hsp33-TrypOx showed a similar destabilization shift upon oxidation as wild type Hsp33 (Figure 5B).

**FIGURE 6:**
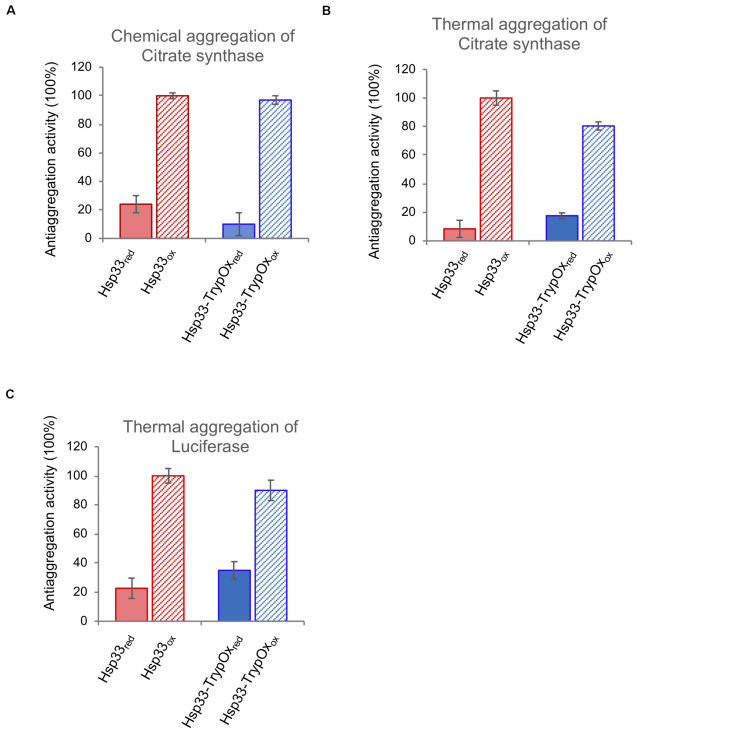
Hsp33 and Hsp33-trypOx are redox-regulated chaperones. The anti-aggregation activity of reduced (solid bars) and oxidized (striped bars) wild type (red) and Hsp33-TrypOx (blue) proteins. The chaperones were added in 4-fold molar excess into either chemically **(A)** or thermally (43°C) **(B)** unfolded Citrate Synthase (75 nM) or **(C)** thermally unfolded luciferase (150 nM) at 43°C. The relative activity was calculated according to the chaperone activity of the oxidized wild type Hsp33.

Next, we examined the ability of the reduced and oxidized variants to prevent aggregation of two common client proteins, luciferase (Luc) and citrate synthase (CS), which were either thermally (CS and Luc) or chemically unfolded (CS). As shown in Figure 6, both proteins showed similar, oxidation-dependent anti-aggregating activity, regardless of substrate type or mode of unfolding. Given the fact that Hsp33 activity is allowed only following oxidation of its C-terminal domain, we can confidently claim that the C-terminal domain of TrypOx is a redox-sensitive domain, with similar structural behavior and ability to alter conformational changes of the adjacent linker region of the bacterial Hsp33 chaperone.

### Redox Status of the TrypOx Redox Domain Mediates Substrate Transfer to the DnaK/J System

The Hsp33 chaperone is an ATP-independent chaperone, which is unable to refold client proteins by itself. The fruitful refolding of the client protein relies on its successful transfer to the ATP-dependent DnaK/J-GrpE system, which requires a reduction of the disulfides in the C-terminal domain of the chaperone, its refolding, and stabilization of the linker domain. These events lead to the destabilization of the client protein bound to Hsp33 and its recognition by the DnaK/J system, which has a higher preference toward fully unfolded proteins than Hsp33 (Reichmann et al., 2012). Hence, the working cycle of Hsp33 is heavily dependent on the reversible thiol oxidation of the C-terminal domain, which mediates open-closed forms of the chaperone along with binding and release of the misfolded substrate.

**FIGURE 7:**
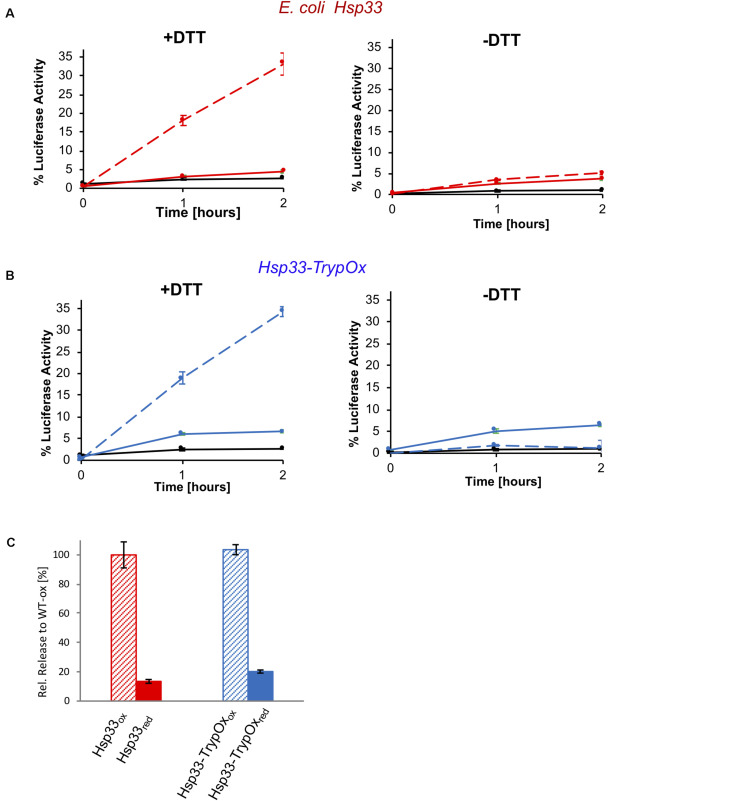
Substrate transfer from Hsp33 and Hsp33-TrypOx to the DnaK/J GrpE system for refolding. Luciferase (150 nM) was thermally unfolded at 43°C in the presence of the **(A)** wild type Hsp33 (red) and **(B)** Hsp33-TrypOx (blue) chaperones (600 nM) for 15 min. After incubation at 25°C for 5 min, the Hsp33-luciferase sample was diluted two-fold, and DnaK (1.5 μM), DnaJ (0.3 μM), GrpE (1.5 μM), 2 mM MgATP in the presence (+DTT) or absence (−DTT) of 2 mM DTT were added to the reaction. Refolding was performed at 25°C for 2 hours and evaluated by recovering luminescence in the presence of 7 μM luciferin and 2 mM MgATP (Turner Biosystems). No refolding of luciferase was observed by DnaK/J, GrpE, or DTT either individually (black curve) or in presence of inactive, reduced Hsp33 and Hsp33-TrypOx proteins (solid line). **(C)** Reduction by DTT and the presence of the oxidized chaperone are required for the successful refolding.

To investigate the redox reversibility of the TrypOx domain, we monitored the reactivation of thermally unfolded luciferase bound to Hsp33 and Hsp33-TrypOx proteins in the presence of chaperone DnaK, its co-chaperone, DnaJ, and the nucleotide exchange factor, GrpE (Figure 7). To generate luciferase-Hsp33 complexes, we incubated luciferase in the presence of either the reduced or oxidized form of the Hsp33 and Hsp33-TrypOx chaperones at 43°C. Luciferase lost most of its activity during the 15 min incubation at heat shock temperatures without the ability to spontaneously refold (Figures 7A,B, black traces). Reduced forms of the chaperones did not form a stable complex with unfolded Luciferase, as previously shown in Figure 6. Therefore, no refolding of luciferase was obtained after adding the DnaK/J/GrpE proteins. However, reduction of the complex formed by oxidized wild type Hsp33 and Hsp33-TrypOx in the presence of the DnaK/J systems restored Luciferase activity to similar levels, demonstrating that both chaperones have comparable substrate transfer efficiency (Figure 7). As expected, a lack of DTT or the DnaK/J proteins did not facilitate luciferase refolding (Figures 7A,B right plot), indicating a need for the reversibility of the redox status of C-terminal domains to obtain fruitful substrate release.

Outcomes of the chaperone and substrate release activity show that *T. brucei* TrypOx’s C-terminus is a modular redox switch that can render the protein function depending on its oxidation status. Hence, it is tempting to speculate that this domain is essential for TrypOx activity in *T. brucei* during oxidative stress conditions.

## Discussion

### Hsp33 Is a Highly Conserved Chaperone

Redox-regulated chaperone Hsp33 was experimentally shown to serve as a first line of defense chaperone during oxidative stress in pathogenic bacteria (*E. coli* and *V. cholera*), and algae *C. reinhardtii* (Segal and Shapira, 2015). Here, we explored sequence conservation of Hsp33 among the tree of life and revealed phylogenetically extended sequence conservation from the bacteria class (harboring more than 1500 homologs) to eukaryotes. Remarkably, eukaryotic homologs are dominantly found in unicellular pathogens (e.g., *Trypanosoma*, *Leishmania*, *Phytophthora*, etc.) or species that live in extreme environmental conditions (*Galdieria*, *Phaeodactylum*, etc.). It’s tempting to speculate that Hsp33 might be beneficial for the pathogenic life cycle by enabling pathogens to cope with changes in environment or the host’s defense system, which applies oxidants to kill pathogens. We were intrigued by this potential modular feature of Hsp33, where the C-terminal domain serves as a redox sensor which can be fused with a non-Hsp33 functional domain (Zhao et al., 2011) as well as replaced between the species as was shown in this study. Therefore, we conducted a bioinformatic analysis which included a motif search using a PSSM matrix derived from homologs of each domain separately, in order to investigate the conservation of the Hsp33 domains separately and together. This analysis revealed that only a few protein families include fused non-Hsp33 functional domains (Supplementary Figure S1), while no family with only the redox domain or other domains was discovered. Thus, Hsp33 evolved as an intact protein, preserving a few sequencing motifs (Figure 1) along with the entire protein, where one region affects the other. The phylogenetic analysis of Hsp33 homologs revealed complete depletion of Hsp33 in archaeal genomes. *Archaea* are a phylogenetically unique domain, which combine properties of eukaryotes and prokaryotes. A majority of the archaeal species do not contain DnaK/J homologs, which may be an acceptor of Hsp33 client proteins. This finding suggests potential co-evolution of DnaK/J-Hsp33 chaperones as seen in bacteria and algae (Segal and Shapira, 2015).

### The Hsp33 Homolog in Trypanosoma Brucei, TrypOx, Is an Important Player in Antioxidant Attack

Although numerous studies have provided evidence of how *Trypanosoma* protects itself during oxidative stress, very little information exists regarding the chaperone and chaperone networks which orchestrate this antioxidant activity.

A majority of studies addressing the stress response of *Trypanosoma* and *Leishmania* pathogens focus on canonical chaperones such as Hsp70, Hsp40, and sHSP, which share sequence and structural similarity with the hosts’, making them a problematic drug target. Here, we identify a new player in the chaperone system, which defends *T. brucei* against oxidative stress, mimicking physiological conditions applied by the host’s immune system. Despite high conservation in bacteria and kinetoplastids, Hsp33 is not found in higher eukaryotes, which makes it a promising drug target against different *Trypanosoma* and *Leishmania* species.

Here, we showed that depletion of the *T. brucei* Hsp33 homolog, TrypOx, leads to decreased sensitivity of procyclic *T. brucei* cells in mild oxidative conditions, which almost do not affect the proteome profile or survival. Proteomic analysis revealed that a combination of mild oxidative stress and a decrease in TrypOx levels alters the antioxidant and chaperone systems, similar to the outcomes of Hsp33 knockout in bacteria (Winter et al., 2005, 2008; Wholey and Jakob, 2012).

Hsp33’s chaperone activity has been very well characterized predominantly in bacteria, as well as in *Chlamydomonas reinhardtii* (Segal and Shapira, 2015). In bacteria and *C. reinhardtii*, site-specific oxidation of the C-terminal domain leads to a cascade of unfolding effects within the Hsp33 protein, leading to its partial unfolding and exposure of client binding regions, preventing aggregation of hundreds of misfolded proteins. Interestingly, algae Hsp33 lost one of its conserved cysteines thus losing Zn-binding activity, making this protein more sensitive to different unfolded conditions including mild heat or oxidation. Recent studies have shown that along with multiple metabolic changes, molecular chaperones and antioxidants are upregulated during different stages in the trypanosome’s life cycle (Koumandou et al., 2008; Mesias et al., 2019). It is therefore worth, in our opinion, continuing to try to map the changes in TrypOx expression during different life cycle stages and environmental conditions, examining its role (if any) in life cell progression and infection.

Intrigued by a potential chaperone activity of TrypOx, we tried several strategies to purify stable forms of TrypOx proteins, including varying bacterial and eukaryotic expression systems, code optimization, refolding from inclusion bodies, fusion with solubility tags, mutagenesis, and more. All these strategies resulted in either completely insoluble or partially insoluble proteins, which aggregated during chaperone assays. Therefore, unfortunately, we cannot determine whether TrypOx has anti-aggregation activity and is able to interact with the *T. brucei* Hsp70/40 system.

Nevertheless, guided by sequence and structural analysis, we decided to utilize the modularity of the C-terminal domain to test TrypOx’s redox-dependent ability to activate chaperone activity mediated by massive structural changes. Activation and client transfer functions of Hsp33 are solely dependent on the redox status of C-terminal domain and are encoded in its unique sequence and structural nature. Replacing the bacterial redox domain with the TrypOx one did not affect the redox-dependent activity of bacterial chaperone regions, resulting in successful anti-aggregation and substrate transfer activities. This suggests that the *T. brucei* and *E. coli* fragments complement each other in a highly orchestrated way, which might mimic the full-length TrypOx activity. This “perfect match” of the bacterial and TrypOx domains is very unique, as bacterial Hsp33 is a highly dynamic protein, mutation of which leads to either no or constitutive activity. Therefore, wild type-like redox-regulated chaperone and transfer activity can be obtained only by correct interactions between C-terminal, linker, and N-terminal domains (Rimon et al., 2017).

Sequence analysis of the *Trypanosoma* and *Leishmania* orthologs showed a ∼100–150 amino acid region only harbored by these species, adjacent to the C-terminal domain. Structural predictions of these sequences point to potential helical and coil features, which might suggest structural plasticity of this adjacent, taxonomically unique fragment. There is a possibility that this region has a unique function i.e., interaction, which is not found in bacterial and other unicellular Hsp33 orthologs, and due to its proximity to the redox domain might be affected by its redox status.

Despite the loss of Hsp33 during the evolution of higher eukaryotes, TrypOx and its orthologs might have clinical and biotechnological value, used as a drug target or in the opposite form, through overexpression which might contribute to enhanced cell growth in oxidative conditions, associated with industrial culture growth.

## Materials and Methods

### *E. coli* and *Trypanosoma brucei* Strains, Growth Conditions and TrypOx Probes

The *E. coli* strains used in this study were Hsp33 knockout (Δ*hslo*) in BL-21 strains. The strains were transformed with pET11a vector containing either wild type *E. coli* Hsp33 or the chimeric Hsp33-TrypOx gene (*E. coli* N-terminus and linker + *T. brucei* C-terminus) (Supplementary Table S4). These strains were grown in LB medium (BD, Cat. 240230) with 100 μg/mL Ampicillin for selection.

Growth experiments in Supplementary Figure S5 were conducted in 96-wells, in M9 medium supplement with either no or 20 μM HOCl on a TECAN plate reader. Change in absorbance (OD600) was monitored overnight at 37°C.

*Trypanosoma brucei* strain 29-13 which carries integrated genes for T7 polymerase and the tetracycline repressor, was grown in SDM-79 medium supplemented with 10% fetal calf serum in the presence of 50 μg/ml hygromycin and 15 μg/ml G418. To establish the cell lines with the dsRNA-expressing construct, transformants were selected with 5 μg/ml phleomycin. The stem-loop structure was prepared using 500 bp amplified from genomic DNA using primer directed to TrypOx (Tb927.6.263) that was cloned into the pZJM vector using three steps (Wang et al., 2000) and integrated into the rRNA locus. The dsRNA production is under the control of the Tetracycline repressor repression. The transfected cells were cloned as previously described (Mandelboim et al., 2003).

Northern analysis-Total RNA was prepared with TRIzol reagent (Sigma), and 20 μg/lane was fractionated on a 1.2% agarose, 2.2 M formaldehyde gel. mRNAs were detected with randomly labeled probes (Random Primer DNA Labeling Mix, Biological Industries Co.).

These strains were grown at 27°C in SDM-79 medium supplemented with 10% fetal calf serum, 1% Penicillin-Streptomycin (Biological industries, Cat. 03-031-1), 6 mg/mL HEPES, 2.2 mg/mL Sodium bicarbonate and 5 μg/mL Hemin, pH 6.9 in the presence of G418 (20 μg/mL) and hygromycin (50 μg/mL) antibiotics for strain selection. For growth curves experiments, cultures were then diluted daily to 10^6^ cell per mL, and cells were counted manually under light microscopy (x40). The tetracycline (20 μM) for TrypOx RNAi induction and hydrogen peroxide (as stated in the figures) were added daily.

### Bioinformatical Analysis of the Hsp33 Protein Family

The PSI-BLAST algorithm (Schaffer et al., 2001) were used to retrieve Hsp33 homologs using two known templates for Hsp33 chaperone sequences, from *E. coli* and *B. subtilis*. The search was done on the NCBI-NR protein database, using a cutoff of 0.001. The fragmented sequences were removed, and sequences with similarity greater than 85% using the CD-HIT software (Li and Godzik, 2006), to leave 1579 sequences. To analyze domain conservation of Hsp33 (the N-terminus, the linker region and the C-terminal domains), multiple sequence alignment using Clustal Omega (Sievers et al., 2011) were performed and then sequence blocks corresponding to N-terminal, linker and C-terminal regions based on the *E. coli*-Hsp33 sequence were extracted. The following borders were used to define the domains: N-terminal domain is between 1 and 173aa; the linker is between: 174 and 226aa, and the C-terminal domain is between 227 and 292aa. These domain-related sequence blocks were used to search for homologous domains in the NCBI database by using the CYRCA (Kunin et al., 2001) and the MAFFT (Katoh et al., 2002) software. No protein families harboring only one of these domains ware detected. However, we did identify 48 proteins harboring all three domains at various levels of conservation, fused to additional functional domains (Supplementary Figure S1).

To calculate the distribution of amino acid conservation among Hsp33 homologous sequences (Figure 1C), we used sequences with 75% identity and lower. Multiple sequence alignment (MSA) was calculated using MAFFT and truncated to positions 200–450, trimming the parts of the MSA before and after the *E. coli* sequence. The distribution of amino acids for each position in the MSA was then calculated and positions appearing as gaps in more than 90% of the sequences were discarded. Each position was given a so-called “conservation score,” consisting of the frequency of appearance of the most prevalent amino acid. The distribution of conservation scores in each domain is presented as a histogram, where each bin consists of a 10% window (e.g., one bin consists of conservation scores between 60 and 70%).

### Sample Preparation for Mass Spectrometry

The *T. brucei* TrypOx RNAi cells were cultivated at 27°C in SDM-79 medium in absence or presence of 20 μM tetracycline for 3 days. Hydrogen peroxide (50 μM) was added, and the cells were incubated at 27°C for 3 h. The cells (about 1 × 10^18^) were then re-suspended in cold PBS, twice. Cells were then lysed in 100 μL of lysis buffer (10 Mm Tris–HCl PH 7.5, 0.5 μl Triton 100%, 1% SDS, 1 mM AEBSF, and 2 μL Protein Inhibitor cocktail (Roche, Cat.11697498001) and incubated on ice for 15 min. The lysate was then centrifuged at 13,000 × *g* for 30 min at 4°C, and supernatants were collected. Samples were diluted using 400 μL of the Urea buffer (8 M urea in 0.1M Tris HCl pH 8.5), and incubated with 10 mM DTT for 1 h, 550 rpm at RT, followed by the standard FASP protocol (Wisniewski et al., 2009). Briefly, the samples were loaded onto the 30 MW amicon tubes, centrifuged for 10 min at 12,000 × *g*. The thiol alkylation was done in the amicon tubes with 50 mM iodoacetamide dissolved in the Urea buffer for 60 min, in dark, under constant agitation (350rpm, 25°C). The proteins were washed three times with the Urea buffer, then twice with the digestion buffer (10% ACN, 25 mM Tris HCl pH 8.5) and centrifuged at 12,000 × *g* for 8 min. The digestion was carried out in a new amicon tube with 1 μL mass spectrometry grade Trypsin (Promega) in 300 μL of digestion buffer and), overnight at 350 rmp, 37°C. Then, after 8 min centrifugation at 12,000 × *g*, the peptides were desalted using in-house C18 Stage Tips as described in (Radzinski et al., 2018).

### Nano-LC-MS/MS Analysis

The peptides were injected and separated by the C18 EasySpray column (Thermo Fisher Scientific) (50 cm, 100 μm IDx2 cm, 100Å, PepMap100 C18, 5 μm, 100Å) at flow 300 ul/min using a Waters Nano-HPLC system (Thermo Fisher Scientific) coupled online to Orbitrap Mass spectrometer, Q Exactive Plus (Thermo Fisher Scientific). To separate the peptides, the column was applied with a linear gradient with a flow rate range from 200 nl/min – 300 nl/min at 35°C: from 1 to 28% in 150 min at flow 200 nl/min, from 28 to 50% in 20 min at flow 200 nl/min, from 50 to 71% in 5 min at flow 300 nl/min, and held at 71% for an additional 15 min, and then equilibrated at 1% for 10 min (solvent A is 0.1% formic acid, and solvent B is 80% acetonitrile, 0.1% formic acid). The Q Exactive was operated in a data-dependent mode. The survey scans (380–2,000 m/z, with a resolution of 70,000 at m/z). The maximum of 12 most abundant isotope patterns with a charge of ≥2 and less than 7 were subjected to higher-energy collisional dissociation with a normalized collision energy of 25, an isolation window of 1.6 m/z, and a resolution of 17,500 at m/z. The MS/MS scans were acquired at a resolution of 17,500 (target value 5E4 charges, maximum ion injection times 57 ms). Dynamic exclusion was 60 s. Data were acquired using Xcalibur software (Thermo Fisher Scientific). To avoid a carryover of the peptides between the samples, the column was washed with 80% acetonitrile for 40 min.

### Data Analysis and Statistics

For protein identification and quantification, we used MaxQuant software (Cox and Mann, 2008), version 1.5.3.30. We used Andromeda search engine incorporated into MaxQuant to search MS/MS spectra against the UniProtKB database of the Trypanosoma Brucei proteome, (Uniprot release, 2005). Enzyme specificity was set to trypsin, allowing cleavage N-terminal to proline and a maximum of two miscleavages. Peptides had to have a minimum length of seven amino acids to be considered for identification. Carbamidomethylation was set as a fixed modification, and methionine oxidation was set as a variable modification. A false discovery rate of 0.05 was applied at the peptide and protein levels. Initial precursor mass deviation till 4.5 ppm and fragment mass deviation till 20 ppm was allowed.

Only proteins identified by more than two peptides were considered. To quantify changes in protein abundance, we utilized the LFQ using the MaxQuant default parameters (Cox and Mann, 2008).

For statistical and bioinformatic analysis, as well as for visualization we used Perseus software (Tyanova et al., 2016)^1^. For functional enrichment analysis, the DAVID webserver (Jiao et al., 2012) was used.

### Purification of the Wild Type Hsp33 and *E. coli*-TrypOx Proteins

The wild type Hsp33 and the *E. coli*-TrypOx chimeric protein were expressed using the *E. coli BL21 ΔhslO* bacteria strain, under the ampicillin resistance. The cells were grown to OD600 of 0.5–0.6 in 4 liters of LB media at 37°C. Expression was induced by 1 mM IPTG, overnight at 18°C. Then, cells were harvested, resuspended in 40 ml of lysis buffer (40 mM HEPES, 500 mM NaCl, and 5 mM imidazole, pH 7.5; and supplemented with 0.2 mg/ml lysozyme, 1 mM AEBSF, and one tablet of Roche’s EDTA-free protease inhibitor cocktail). After 30 min of incubation on ice, the cells were disrupted at 25,000 psi and the lysate was centrifuged for 45 min at 14,500 rpm in 4°C.

The proteins were purified on Anion exchange chromatography by using three 5 ml HiTrap Q HP columns (GE Healthcare) equilibrated with 5 column volumes (CV) of the buffer (40 mM Hepes-KOH, 200 mM KCl, pH 7.5). Fractions were collected and dialyzed overnight against 5 mM KH_2_PO4 pH 6.8. Then the protein sample was applied to the self-packed, 58 ml hydroxyapatite column (hydroxyapatite resin from BioRad). Proteins were separated by using a 200 ml gradient of 5–70 mM KH_2_PO4 pH 7.5, and dialyzed overnight against 40 mM KH_2_PO4, 200 mM KCl, pH 7.5. Final purification was done by SEC (HiLoad 16/60 Superdex 75 PG, GE Healthcare).

Protein purity was determined and confirmed by SDS-PAGE, and protein concentration was determined by light absorbance at 280 nm.

Protein reduction and oxidation was done as described in Rimon et al. (2017); Fassler et al. (2018).

### Far-UV CD Spectroscopy

To determine changes in the secondary structure of the wild type and *E. coli*-TrypOx protein, far-UV CD spectra were recorded for 190–260 nm, by using the Jasco J810 spectropolarimeter. For the CD spectra and thermostability measurements, we used 5 μM TrypOx proteins in 40 mM KH_2_PO4, pH 7.5 in a quartz cell with a 1 mm path length at 25°C. All spectra were corrected according to a 40 mM KH_2_PO4 pH = 7.5 blank solution. To determine the thermostability of the mutants, the CD signal at 218 nm was measured while raising the temperature from 20 to 80°C at a rate of 1°C/min.

## Data Availability Statement

The original contributions presented in the study are publicly available. This data can be found here: https://www.ebi.ac.uk/pride/PXD018965.

## Author Contributions

SA, RF, VC, MG, TA, LK, OR, SM, and DR designed the research and related the experiments. VC, LK, and SM established the RNAi TrypOx lines and tested them by NB. RF established the *T. brucei* culture work in the Reichmann lab and conducted the growth experiments. SA performed the proteomic analysis as verified the growth phenotypes in different RNAi TrypOx strains. TA, OR, and DR performed the bioinformatic analysis of the Hsp33 protein family. MG and DR designed the TrypOx chimeric proteins. MG expressed and purified the chaperones as well as conducted the CD, chaperone and the client transfer assays. MG and OR conducted survival growth curve of Hsp33-TrypOx containing construct. SA and DR wrote the manuscript while RF and SM assisted and edited the manuscript. All authors contributed to the article and approved the submitted version.

## Conflict of Interest

The authors declare that the research was conducted in the absence of any commercial or financial relationships that could be construed as a potential conflict of interest.

## Footnotes

1 http://www.coxdocs.org/doku.php?id=perseus:start
