# Supplementary material for: TrypOx, a Novel Eukaryotic Homolog of the Redox-Regulated Chaperone Hsp33 in Trypanosoma brucei
Source: Front Microbiol. 2020 Aug 6;11:1844. doi: 10.3389/fmicb.2020.01844 (PMC7423844; doi:10.3389/fmicb.2020.01844)
Supplement: Supplementary file 1 [file Image_1.pdf]

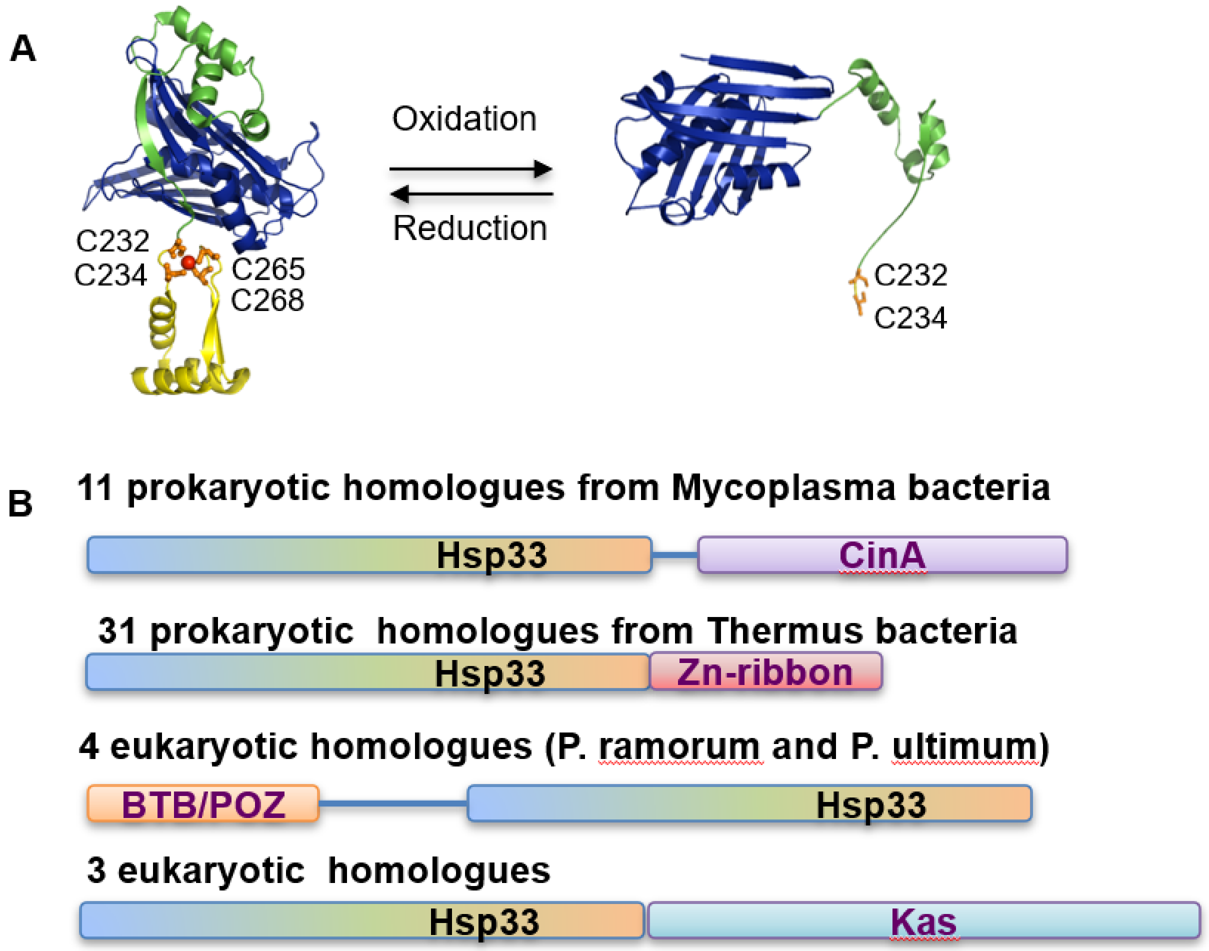

Figure S1. The domain architecture of Hsp33 homologues. (A) Activation of Hsp33 by oxidation leads to conformational changes. The Hsp33 architecture consists of a thermostable N-terminus (blue), a thermobile linker (green), and the C-terminal (yellow) regions. Activation of Hsp33 by oxidation leads to the formation of two disulfide bonds between Cys232 and Cys234, and Cys265 and Cys268, which in turn leads to the unfolding of the redox domain and destabilization of the linker region (green). For representation we used reduced, Bacillus subtilis Hsp33 X-ray structure (PDB: 1VZY) and oxidized E.coli Hsp33 X-ray structure (PDB: 1HW7). Due to high flexibility of the C-terminal domain, its structure in the oxidized form was not solved by X-Ray crystallography (B) The architecture of Hsp33 homologues harboring additional functional domains as defined by CD database (NCBI).
