# Supplementary material for: TrypOx, a Novel Eukaryotic Homolog of the Redox-Regulated Chaperone Hsp33 in Trypanosoma brucei
Source: Front Microbiol. 2020 Aug 6;11:1844. doi: 10.3389/fmicb.2020.01844 (PMC7423844; doi:10.3389/fmicb.2020.01844)
Supplement: Supplementary file 2 [file Image_2.pdf]

Figure S2

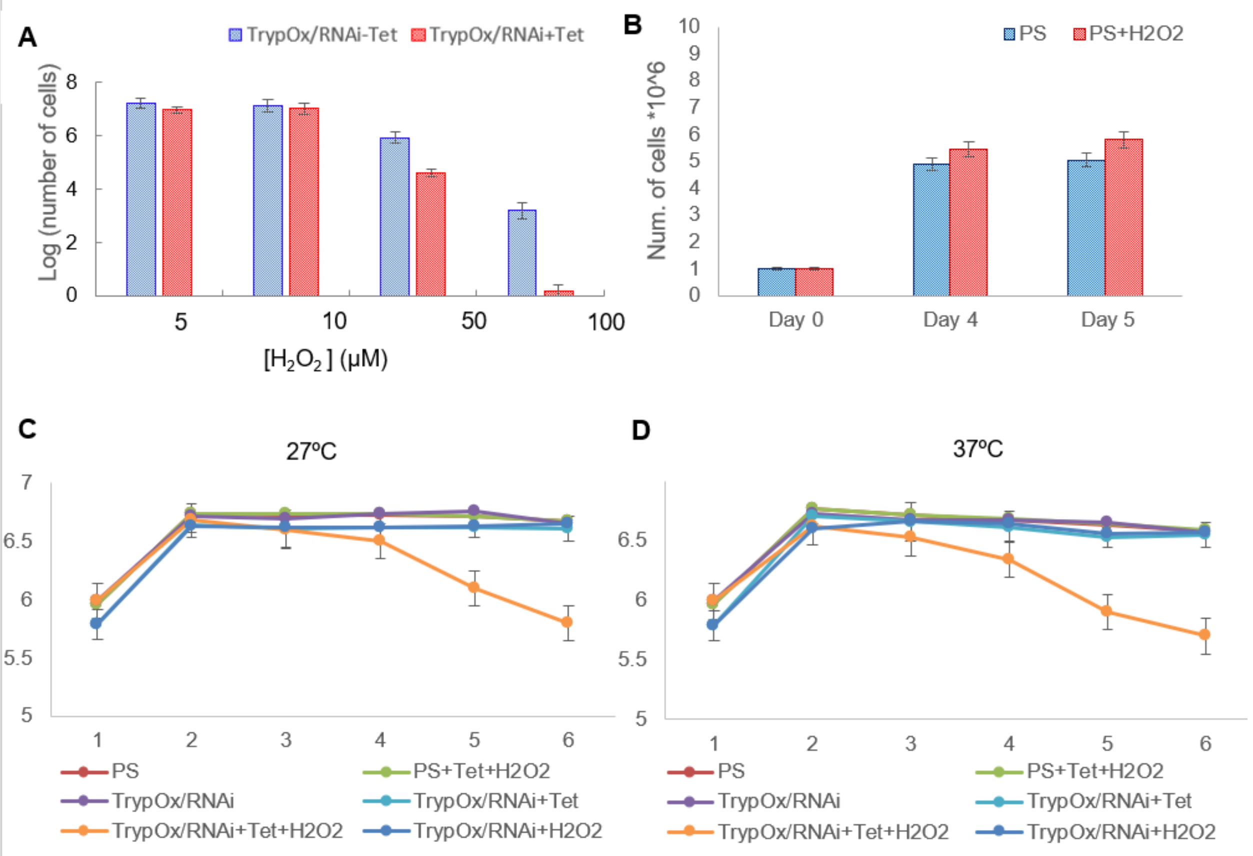

Figure S2. Oxidation-dependent growth of the TrypOx RNAi strain. (A) Survival of induced (red) and non-induced (blue) cells expressing TrypOx RNAi in the absence of varying H_2O_2 concentrations. (B) Survival of the PS cells in the presence (red) and absence (blue) of 50µM H_2O_2 over the course of 5 days. (C-D) The sensitivity of parental and TrypOx RNAi strains to 50 µM H_2O_2 at (C) 27°C and (D) 37°C. Tetracycline induction did not alter the growth in both strains. Growth of the parental strain was not affected by the supplement of peroxide in the absence and presence of tetracycline.
