# Supplementary material for: TrypOx, a Novel Eukaryotic Homolog of the Redox-Regulated Chaperone Hsp33 in Trypanosoma brucei
Source: Front Microbiol. 2020 Aug 6;11:1844. doi: 10.3389/fmicb.2020.01844 (PMC7423844; doi:10.3389/fmicb.2020.01844)
Supplement: Supplementary file 3 [file Image_3.pdf]

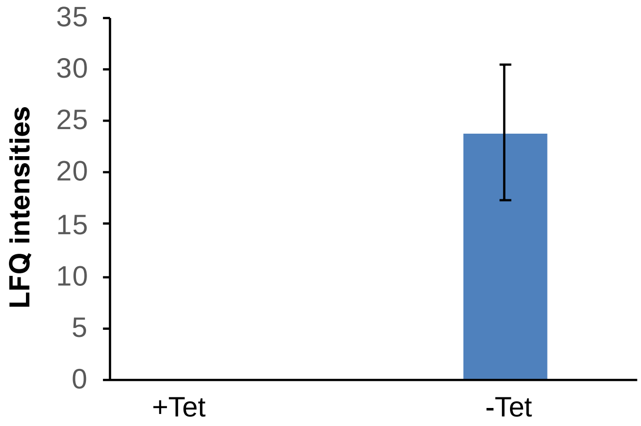

Figure S3. Silencing of TrypOx transcript resulted in no protein synthesis. TrypOx protein intensities TrypOx/RNAi strains in absence (uninduced) and presence (induced) tetracycline. Protein identification and intensities were measured using LC-MS/MS in three biological replicates.
