# Supplementary material for: TrypOx, a Novel Eukaryotic Homolog of the Redox-Regulated Chaperone Hsp33 in Trypanosoma brucei
Source: Front Microbiol. 2020 Aug 6;11:1844. doi: 10.3389/fmicb.2020.01844 (PMC7423844; doi:10.3389/fmicb.2020.01844)
Supplement: Supplementary file 4 [file Image_4.pdf]

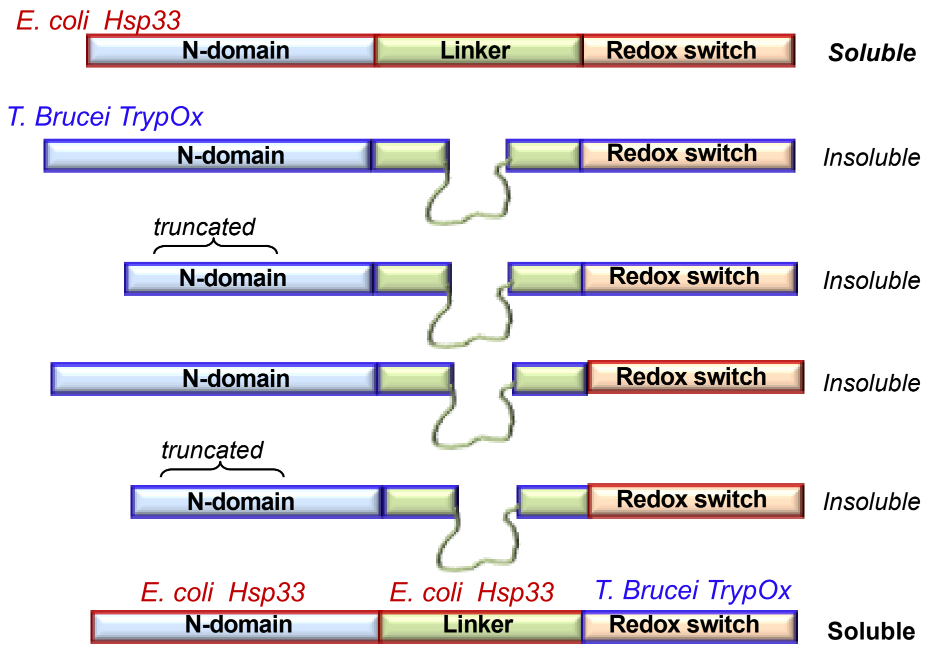

Figure S4. Schematic representation of varying chimeric proteins consisting of Hsp33 and TrypOx domains. To overcome the insolubility of the full-length TrypOx protein, we utilized the modular structure of bacterial Hsp33 and attempted to express and purify proteins harboring both Hsp33 (red frame) and TrypOx (blue frame) domains. Only one chimeric protein, comprised of N-terminal and linker bacterial regions fused with the TrypOx redox domain was soluble.
