# Supplementary material for: TrypOx, a Novel Eukaryotic Homolog of the Redox-Regulated Chaperone Hsp33 in Trypanosoma brucei
Source: Front Microbiol. 2020 Aug 6;11:1844. doi: 10.3389/fmicb.2020.01844 (PMC7423844; doi:10.3389/fmicb.2020.01844)
Supplement: Supplementary file 5 [file Image_5.pdf]

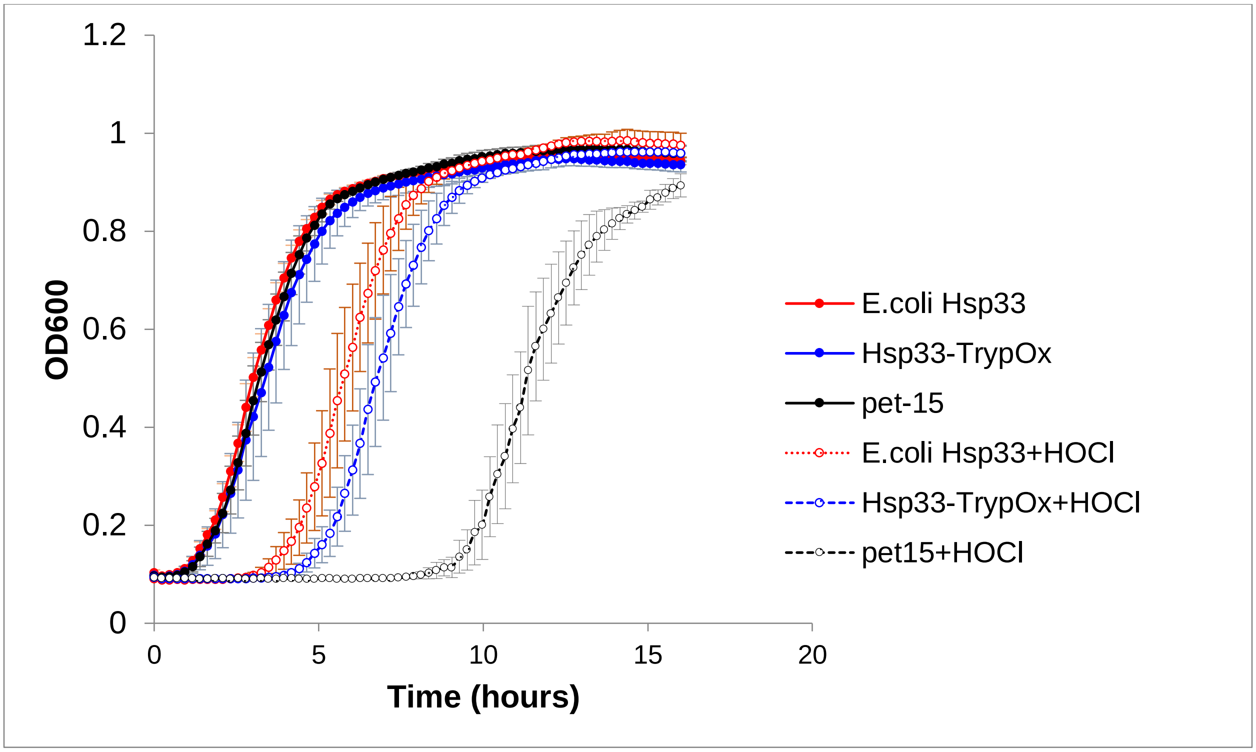

Figure S5. Survival of BL21ΔhslO cells expressing either wild type *E.coli* Hsp33 (red) or chimeric Hsp33-TrypOx (blue) or no protein (black) at absence (solid) or presence (dashed) of 20 μM HOCl. Three-four biological replicates were used in this analysis.
