# Supplementary material for: TrypOx, a Novel Eukaryotic Homolog of the Redox-Regulated Chaperone Hsp33 in Trypanosoma brucei
Source: Front Microbiol. 2020 Aug 6;11:1844. doi: 10.3389/fmicb.2020.01844 (PMC7423844; doi:10.3389/fmicb.2020.01844)
Supplement: Supplementary file 6 [file Table_1.pdf]

Table S1. Protein homologues with an additional non-Hsp33 domain

Taxonomy	Additional domain	GI (NCBI) number
Mycoplasma ovipneumoniae	competence-damaged protein, CinA	498006943
Mycoplasma flocculare	competence-damaged protein, CinA	488621072
Mycoplasma dispar	competence-damaged protein, CinA	765307823
Mycoplasma conjunctivae	competence-damaged protein, CinA	502285817
Mycoplasma hyorhinis	competence-damaged protein, CinA	503067451
Mycoplasma hyopneumoniae	competence-damaged protein, CinA	499519431
Phytophthora palmivora	BTB like domain (DUF3342 domain)	1338257754
Phytophthora cactorum	BTB like domain (DUF3342 domain)	1418029886
Phytophthora kernoviae	BTB like domain (DUF3342 domain)	1492271495
Nothophytophthora	BTB like domain (DUF3342 domain)	1492297074
Achlya hypogyna	Kinesin-associated protein (KAS)	1173942638
Thraustotheca clavata	Kinesin-associated protein (KAS)	1173948825
Aphanomyces astaci	Kinesin-associated protein (KAS)	1475206491
Thermus thermophilus	Zn ribbon domain	499486343
Thermus aquaticus	Zn ribbon domain	489135825
Oceanithermus profundus	Zn ribbon domain	503223216
Thermus scotoductus	Zn ribbon domain	505921673
Marinithermus hydrothermalis	Zn ribbon domain	503469464
Thermus sp. CCB_US3_UF1	Zn ribbon domain	504328475
Thermus oshimai	Zn ribbon domain	511099563
Thermus islandicus	Zn ribbon domain	551067784
Thermus antranikianii	Zn ribbon domain	655044257
Thermus tengchongensis	Zn ribbon domain	740200430
Thermus amyloliquefaciens	Zn ribbon domain	740215007
Thermus filiformis	Zn ribbon domain	740219580
Thermus parvatiensis	Zn ribbon domain	982900045
Thermus arciformis	Zn ribbon domain	1224991293
Thermus brockianus	Zn ribbon domain	1103715456
Thermus tenuipuniceus	Zn ribbon domain	1357954445
Meiothermus	Zn ribbon domain	1402629770
Thermus caldifontis	Zn ribbon domain	1434566446
Thermus sediminis	Zn ribbon domain	1468270746
Thermus tengchongensis	Zn ribbon domain	1608627423
Thermus caldilimi	Zn ribbon domain	1608621924
