# Supplementary material for: TrypOx, a Novel Eukaryotic Homolog of the Redox-Regulated Chaperone Hsp33 in Trypanosoma brucei
Source: Front Microbiol. 2020 Aug 6;11:1844. doi: 10.3389/fmicb.2020.01844 (PMC7423844; doi:10.3389/fmicb.2020.01844)
Supplement: Supplementary file 9 [file Table_4.pdf]

Table S4-A. Strains used in this study

Strain	Genotype	Plasmid	Comment
BL21-*E.coli Hsp33*	*Δhslo,*	Pet11a containing the *hslo* gene, *Amp resistance*	Expression of E.coli Hsp33
BL21-*E.coli -TrypOx*	*Δhslo,*	Pet11a containing the *chimeric Hsp33-TrypOx gene*, *Amp resistance*	Expression of HHsp33sp33-TrypOx protein
T.brucei Trypox RNAi T. brucei	*T. brucei* strain 29-13	pZJM vector with the anti-trypOx fragment	Silencing of TrypOx

Table S4-B. Sequences of primers and proteins used in this study

Name	Sequence
Tb 927.6.2630 Sense	5'-GGGTCTAGACTTTGACGCAGCTCATAGAC-3'
Tb 927.6.2630 antisense	5'-AAAACGCGTAACGCTAAATATTTCAGGTT-3'
BL21-*E.coli -WT Hsp33*	MGSSHHHHHHSSGLVPRGSHMIMPQHDQLHRYLFENFAV RGELVTVSETLQQILENHDYPQPVKNVLAELLVATSLLTAT LKFDGDITVQLQGDGPMNLAVINGNNNQQMRGVARVQGE IPENADLKTLVGNGYVVITITPSEGERYQGVVGLEGDTLAA CLEDYFMRSEQLPTRLFIRTGDVDGKPAAGGMLLQVMPAQ NAQQDDFDHLATLTETIKTEELLTLPANEVLWRLYHEEEVT VYDPQDVEFKCTCSRERCADALKTLPDEEVDSILAEDGEID MHCDYCGNHYLFNAMDIAEIRNNASPADPQVH
BL21-*E.coli -TrypOx*	MIMPQHDQLHRYLFENFAVRGELVTVSETLQQILENHDYP QPVKNVLAELLVATSLLTATLKFDGDITVQLQGDGPMNLA VINGNNNQQMRGVARVQGEIPENADLKTLVGNGYVVITIT PSEGERYQGVVGLEGDTLAACLEDYFMRSEQLPTRLFIRTG DVDGKPAAGGMLLQVMPAQNAQQDDFDHLATLTETIKTE ELLTLPANEVLWRLYHEEEVTVYDPQDVEFKCRCSKNNFL RALVALPEEQLSSLMEETSFRCTFCAKEHVLQPEDWSKLLR DRTSFKK
